# The expression of the Sprouty 1 protein inversely correlates with growth, proliferation, migration and invasion of ovarian cancer cells

**DOI:** 10.1186/1757-2215-7-61

**Published:** 2014-06-08

**Authors:** Samar Masoumi-Moghaddam, Afshin Amini, Anahid Ehteda, Ai-Qun Wei, David Lawson Morris

**Affiliations:** 1 Department of Surgery, St George Hospital, The University of New South Wales, Gray Street, Kogarah, Sydney NSW 2217, Australia; 2 Department of Orthopedic Surgery, St. George Hospital, The University of New South Wales, Gray Street, Kogarah, Sydney NSW 2217, Australia

**Keywords:** Spry1, Ovarian cancer, Invasion, Migration, Proliferation, Survival

## Abstract

**Background:**

Our recent study on a panel of human ovarian cancer cells revealed that SKOV-3 cells barely express the Sprouty isoform 1 (Spry1) while 1A9 cells maintain it at a level similar to normal ovarian cells. Here we investigated the functional outcomes of induced alterations in the expression of Spry1 in the two cell lines in vitro.

**Methods:**

Using the Spry1 specific plasmid and siRNA, the expression of Spry1 was induced and conversely silenced in SKOV-3 and 1A9 cells, respectively. The functional outcome was investigated by means of proliferation, MTT, scratch-wound, migration and invasion assays and selection of the stable clones. Mechanism of the effect was explored by Western blot.

**Results:**

In the Spry1-transfected SKOV-3 cells, a significant reduction in growth and proliferation was evident. Stable clones of the Spry1-transfected SKOV-3 were almost undetectable after day 14. The number of migrated and invaded cells and the percentage of the scratch closure were significantly lower in the Spry1-transfected group. Spry1 silencing in 1A9 cells, on the other hand, led to a significant increase in cell growth and proliferation. The number of migrated and invaded cells and the percentage of the scratch closure significantly increased in Spry1-silenced 1A9 group. Mechanistically, overexpression of Bax, activation of caspases 3, 7, 8 and 9, cleavage of PARP and attenuation of Bcl-2 and Bcl-xl were observed along with reduced activation of Erk and Akt and increased amount and activity of PTEN in the Spry1-transfected SKOV-3 cells.

**Conclusions:**

Here, we report the inverse correlation between the expression of Spry1 and growth, proliferation, invasion and migration of ovarian cancer cells.

## Background

The Sprouty protein family is a downstream modulator of the receptor tyrosine kinase (RTK) signaling and thus a major contributor to the regulation of the eukaryotic cells biology. Up to now, four mammalian isoforms of the protein, designated Spry1-4, have been identified. Spry1, the first isoform to be discovered, was initially introduced as a potent feedback inhibitor of the FGF receptor signaling during the tracheal development of the Drosophila embryos [1]. Owing to their regulatory function, deregulation of Sprouty proteins and its contribution to pathophysiology of cancer have been studied in different malignancies [2]. Carcinomas of the breast [3] and prostate [4], for example, have reportedly been associated with Spry1 downregulation. Preclinically, anti-proliferative and tumor suppressing roles have been described for Spry1 in prostate [4], liver [5] and medullary thyroid cancer cells [6]. Spry1 has also been implicated as a marker of good prognosis in patients with renal cell carcinoma [7]. Our prior work showed that Spry1 is downregulated in the majority of the ovarian cancer cell lines studied [8]. Since there clearly is a need for further investigation exploring the role of Spry1 in the context of ovarian cancer, we examined in the present study the correlation between the expression of Spry1 and the biological behavior of ovarian cancer cells. Using two cell lines with distinct Spry1 expression profiles, here we demonstrate how alterations in the protein expression impact functional properties of ovarian cancer cells.

## Methods

### Cell culture

Human ovarian cancer cell lines SKOV-3 and 1A9, a variant derived from A2780 [9], were obtained from the American Type Culture Collection (ATCC) (Manassas, VA, USA) and were maintained in a humidified 5% CO2 incubator at 37°C in RPMI-1640 (Invitrogen, CA, USA) supplemented with 10% fetal bovine serum (FBS) and 1% penicillin-streptomycin mixture (Invitrogen, CA, USA).

### Transfection and silencing

Electroporation-based transfection and silencing were carried out using 4D-Neucleofector System and SF Cell Line 4D-Neucleofector Kit (Lonza Group, Basel, Switzerland) according to the manufacturer’s protocol after initial optimization for the cell lines. The two negative controls designed for the transfection experiments included “-vector” (plasmid-free) and “+vector” (vector only) transfection groups. For transient transfection, SKOV-3 cells were washed with PBS, detached with trypsin and suspended in the supplemented Nucleofector Solution SF (2 × 10^6^/100 μl). After adding the pcDNA3.1/Spry1 construct and pcDNA3.1 empty vector (Invitrogen, Life Technologies, CA, USA) to the Spry1-transfection and + vector samples, respectively, all samples were transferred into the 4D-Neucleofector device and the reaction was run with the optimized program (pulse code: EH-100). Samples were then transferred to the culture vessels and cultured for further analysis. For the stable transfection experiment, exposure to the selection condition (culture media containing G418-Geneticin (Gibco, Life Technologies, CA, USA) at a final concentration of 300 μg/ml) for selection of the stably-transfected clones was started two days post-transfection. On day 14 post-selection, Geneticin-resistant clones were fixed with 100% methanol and stained with crystal violet. Transient silencing of Spry1 in 1A9 cells was performed using Spry1 Pre-design Chimera RNAi (Abnova, Taiwan). Reaction was similarly carried out using 4D-Neucleofector System set up for the cell line (pulse code: EN-138). Efficiency of the electroporation was evaluated by visualization of the green fluorescent protein (GFP) encoded by the co-transfected control plasmid (pmaxGFP Vector (Lonza Group, Basel, Switzerland)) showing transfection efficiency of >80%, as well as by western blot analysis of the Spry1 expression in the control transfected or silenced cells. The specificity of the constructs and plasmids were confirmed by western blot as the expression of other members of the Sprouty family was found unaffected.

### Western blotting

At the endpoints, cultured cells were homogenized in a protein lysis buffer (RIPA buffer) containing 10% protease inhibitor (Sigma-Aldrich, Missouri, USA) and the protein concentrations were quantified by BioRad protein assay (Bio-Rad, CA, USA). Then, the same amounts of the proteins were separated by sodium dodecyl sulfate-polyacrylamide gel electrophoresis, transferred to PVDF membranes (Millipore Corporation, MA, USA). The following primary antibodies were then applied to the membranes according to the manufacturers’ protocols: rabbit polyclonal anti-caspase 3, anti-Bcl2 (Santa Cruz Biotechnology, Santa Cruz, CA, USA), anti-caspase 8 (R&D Systems, Minneapolis, MN, USA), anti-caspase 9, anti-PARP, anti-Akt, anti-Phospho-PTEN, rabbit monoclonal anti-caspase 7, anti-Bcl-xl, anti-Bax, anti-phospho-Akt, anti-ERK, anti-phospho-ERK, anti-PTEN (Cell Signaling Technology Inc, Danvers, MA, USA), and mouse monoclonal anti-Spry1 (Abnova, Taiwan). The membranes were washed and treated with appropriate horseradish peroxidase-conjugated secondary antibodies (Cell Signaling Technology Inc, Danvers, MA, USA). Similar process was carried out for the GAPDH protein, as a loading control, using 1:20000-diluted anti-GAPDH mouse monoclonal antibody (Sigma-Aldrich, Missouri, USA). The antigen-antibody reaction was digitized with ImageQuant LAS 4000 Biomolecular imager and ImageQuant software (GE Healthcare, UK).

### MTT assay

Appropriate number of the Spry1-transfected SKOV-3 cells and Spry1-silenced 1A9 cells along with equal number of their respective controls were cultured in 96-well plates at 37°C in 5% CO2 incubator for 24, 48 and 72 hours. At the endpoints, cells were incubated with Thiazolyl Blue Tetrazolium Bromide (Sigma-Aldrich, Missouri, USA) at a concentration of 0.5 mg/ml for further 4 hours. Resulting formazan crystals were dissolved with 100 μl of dimethyl sulfoxide (DMSO) and absorbance was read using PowerWaveX microplate reader (Bio-Tek Instruments Inc, VT, USA) at the working wavelength of 562 nm.

### Trypan blue assay

Appropriate number of the Spry1-transfected SKOV-3 cells and Spry1-silenced 1A9 cells along with equal number of their respective controls were cultured in 6-well plates. At 24 h, 48 h and 72 h after plating, cells were trypsinized and resuspended in medium. Cell suspensions were then diluted 1:10 in trypan blue and the actual number of the cells was calculated using a hemocytometer.

### Scratch assay

Control, Spry1-transfected and Spry1-silenced cells were grown to confluence and the cell monolayers were scraped with the tip of a pipette to create a uniform scratch. The culture media were then replaced with fresh media supplemented with 2% FBS and the reference points for imaging were marked. Using Leica DM IRB microscope equipped with Leica DC200 camera and IM50 software (Leica Microsystems, Germany), plates were viewed with a 5X objective and sequential imaging was performed at the given time points. Images were then analyzed with ImageJ software (Research Services Branch, National Institutes of Health, USA) and the results were quantified by measuring the percentage of the scratch closure.

### Cell migration and invasion assays

24-well Transwell system with polycarbonate membranes of 8 mm pore size was used for cell migration and invasion assays. Briefly, 24 hours post transfection, appropriate amount of cell suspensions (5 *×* 10^4^ SKOV-3 cells or 1 *×* 10^5^ 1A9 cells in 500 μl of 0.1% BSA/RPMI per well) was transferred to the upper compartments of the Boyden chambers either coated with matrigel (BD Biosciences, NJ, USA) for invasion assay or without matrigel coating (Corning Life Sciences, MA, USA) for migration assay. Lower compartments were filled with 750 μl of the same medium supplemented with 10% FBS. Cells were then allowed to migrate and invade at 37°C. At the given time points, content of the upper compartment was discarded and upper surface of the membrane was wiped with a cotton swab. Cells on the lower surface of the membrane were fixed in 100% methanol, stained with Giemsa and counted under the light microscope in at least eight different fields across the membrane.

### Statistical analysis

All data presented are representative of three independent experiments performed in triplicate. Statistical analysis was conducted using GraphPad InStat (GraphPad Prism 6, San Diego, California, USA). Student’s *t*-test was applied for unpaired samples and *P* values *<* 0*.*05 were considered significant. Since no significant difference was found between the data from + vector and -vector controls in the experiments with transiently-transfected SKOV-3, +vector was considered as their negative control for the statistical analysis.

## Results

### Induced expression of Spry1 is deleterious for viability of the ovarian cancer cell line SKOV-3

**Figure 1:**
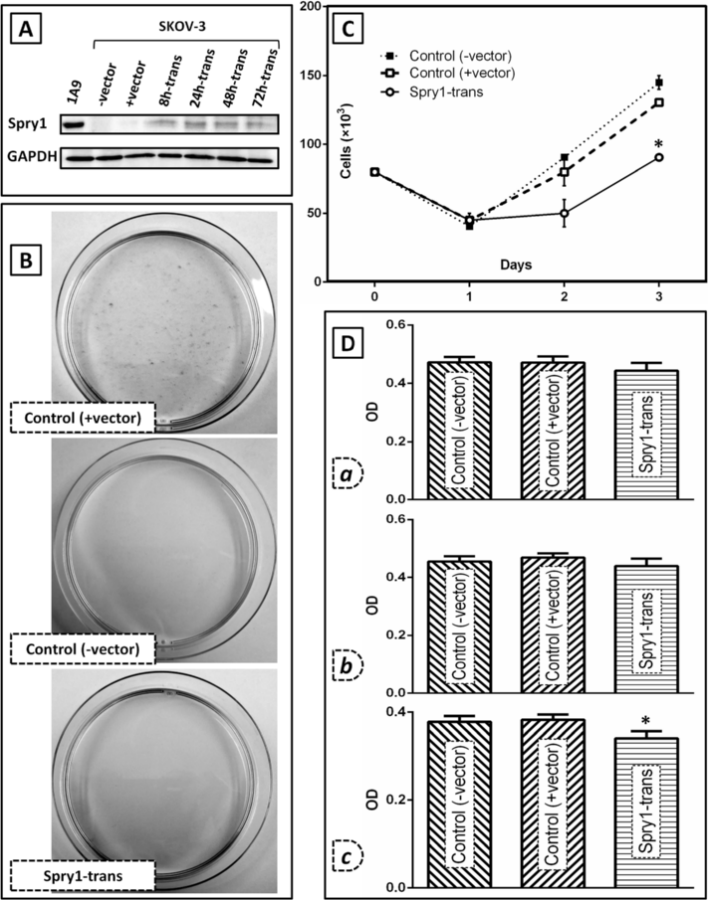
**Effect of the induced expression of Spry1 on cell viability in SKOV-3 human ovarian cancer cells as compared to the negative controls.** The two control groups include the SKOV-3 cells transfected with pcDNA3.1 plasmid (+vector, Genticin resistant) and the SKOV-3 cells with no plasmid transfection (−vector, Genticin sensitive). **A**. Western blot analysis of the protein expression in the Spry1-transfected cells as compared to the controls. Results show expression of Spry1 examined at 8 (8 h-trans), 24 (24 h-trans), 48 (48 h-trans) and 72 hours (72 h-trans) post-transfection. The Spry1-expressing cell line 1A9 was used as a positive control for the protein expression. GAPDH blot is shown as the loading control of the experiment. **B**. Crystal violet staining of the Spry1-transfected, +vector and -vector SKOV-3 cells after 2 weeks under Geneticin selection. As seen, no colony was formed by the Spry1-transfected and -vector cells. **C**. Trypan blue assay and; **D**. MTT assay, on Spry1-transfected cells on days 1 *(a)*, 2 *(b)* and 3 *(c)* post transfection as compared to the negative controls. MTT assay cell viability results are shown as optical density (OD) units which are linearly correlated with the cell number. Both assays indicated a significant decrease in growth and proliferation of the Spry1-transfected cells on day 3 post-transfection. Images are representative of three independent experiments. Data are shown as mean ± SE. Significant values (<0.05) are marked by asterisks.

We previously demonstrated that the human ovarian cancer cell line SKOV-3 barely expresses Spry1 [8]. To assess the effect of the Spry1 expression on ovarian cancer biology, we intended to examine viability of SKOV-3 cells after transfection with the plasmid encoding the full**-**length sequence of Spry1 (Figure 1). Initially, the expression of the Spry1 protein was detected by Western blot at 8, 24, 48 and 72 hours, post-transfection (Figure 1A). Next, the impact of sustained as well as transient expression of the protein on the cell viability was evaluated. In our stable transfection experiment under Geneticin selection for 14 days, no Spry1**-**transfected clones survived whereas numerous colonies of the + vector SKOV-3 formed (Figure 1B). Using the trypan blue (Figure 1C) and MTT (Figure 1D) assays, we also found that the expression of Spry1 resulted in a significant decrease in the growth and proliferation of the transfected cells at 72 hours post transfection (p-values of 0.0003 and 0.0042 for trypan blue and MTT assay, respectively). Taken together, we observed that induced expression of the Spry1 adversely impacts the SKOV-3 cell viability, *in vitro*.

### Spry1 transfection of SKOV-3 cells diminishes migration and invasion

**Figure 2:**
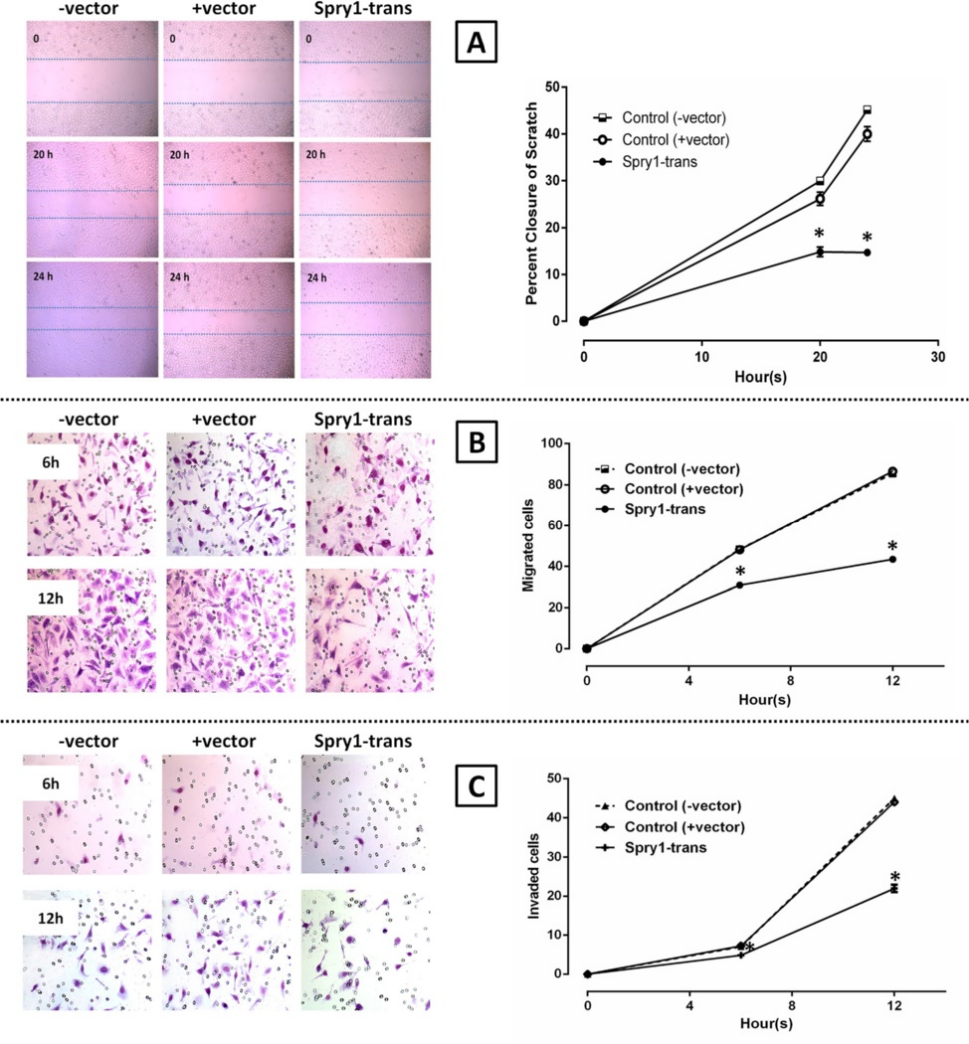
**Spry1-induced inhibition of wound healing, migration and invasion in SKOV-3 cells. A**. Scratch assay imaged at 0, 20 and 24 hours after scratch (left). Data analysis showed a significant decrease in the percent closure by the Spry1 transfection group as compared to the negative controls (+vector and –vector) 20 and 24 hours after scratch (right). **B**. Migration assay using 24-well Transwell system (left), showing a significantly lower number of the migrated cells in the transfection group 6 and 12 hours after plating (right). **C**. Invasion assay using matrigel-coated Transwell plates imaged 6 and 12 hours after plating (left), demonstrating a significantly lower number of the invaded cells in the transfection group assayed at the two endpoints (right). Images are representative of three independent experiments. Data are shown as mean ± SE. Significant values (<0.05) are marked by asterisks.

To investigate the influence of the Spry1 expression on other mitogen-dependent processes, three different assays were employed in the next step to compare the motility and invasion of the Spry1-transfected cells with those of the negative control group (Figure 2). In the scratch assay, the percent closure of the wounded area in the Spry1 transfection group showed a significant decline measured at hours 20 (p-value: 0.0232) and 24 (p-value: 0.0046) after scratch (Figure 2A). Results from the migration assay (Figure 2B) indicated that the number of the Spry1-transfected cells migrated was significantly lower than their control counterparts, 6 (p-value: 0.0090) and 12 hours (p-value: 0.0002) after plating. The invasion assay (Figure 2C) similarly showed reduced number of the invaded cells in the Spry1 transfection group examined at hours 6 (p-value: 0.0159) and 12 (p-value: 0.0005). In sum, induced expression of Spry1 was associated with attenuation of the SKOV-3 cell motility and invasion, *in vitro*.

### Spry1 knockdown enhances growth and proliferation of the 1A9 human ovarian cancer cells

**Figure 3:**
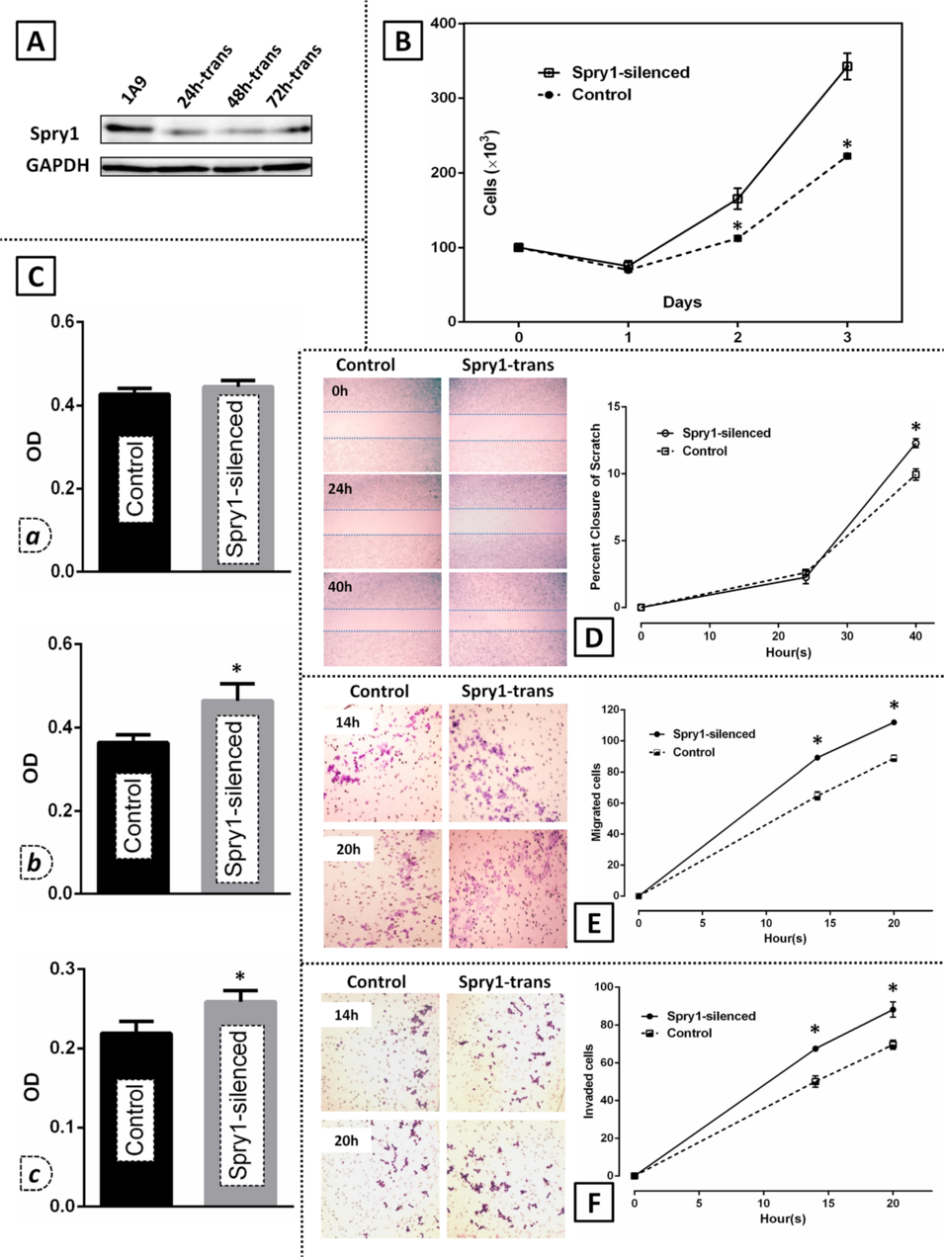
**Effect of the Spry1 knockdown on viability, migration and invasion of 1A9 human ovarian cancer cells. A**. Western blot analysis of the Spry1 expression in 1A9 cells after Spry1 silencing with specific siRNA at 24 h (24 h-trans), 48 h (48 h-trans) and 72 h (72 h-trans) as compared to the control. **B**. Trypan blue assay and; **C**. MTT assay on the Spry1-silenced cells and their control counterparts 24 *(a)*, 48 *(b)*, and 72 hours *(c)* post transfection. MTT assay cell viability results are shown as optical density (OD) units which are linearly correlated with the cell number. Results show a significant increase in the growth and proliferation of the silenced cells at 48 h and 72 h endpoints. **D**. Scratch assay photographed at 0, 24 and 40 hours after scratch (left) shows that the percent closure developed by the Spry1-silenced cells 40 hours after scratch is significantly higher than that observed in the control group (right). **E**. Migration assay imaged 14 and 20 hours after plating (left), showing a significantly higher number of the Spry1-silenced cells migrated at the two endpoints as compared to control (right). **F**. Invasion assay imaged at 14 and 20 hours after plating (left), indicating a significantly higher number of the Spry1-silenced cells invaded at the endpoints as compared to their control counterparts (right). Images are representative of three independent experiments. Data are shown as mean ± SE. Significant values (<0.05) are marked by asterisks.

As we reported previously [8], the human ovarian cancer cell line 1A9 expresses the Spry1 protein. To evaluate how the inhibited expression of the protein could affect the ovarian cancer cell biology, Spry1 was initially silenced in 1A9 cells using the specific siRNA (Figure 3), with the protein expression being examined at 24, 48 and 72 hours post transfection (Figure 3A). Both the Spry1-expressing (control) and Spry1-silenced cells were then assayed for their ability to grow and proliferate. In the trypan blue assay (Figure 3B), the diminished expression of Spry1 in the silenced cells was associated with a significant increase in their growth and proliferation assessed at 48 h (p-value: 0.0365) and 72 h (p-value: 0.0228) endpoints. MTT assay of the cell viability (Figure 3C) returned similar results as enhanced growth and proliferation of 1A9 cells in the Spry1 knockdown group 48 and 72 hours post silencing (p-values of 0.0011 and 0.0024, respectively). Collectively, enhanced viability of 1A9 cells was resulted when the expression of Spry1 was knocked down, *in vitro*.

### Spry1 knockdown in 1A9 cells augments wound healing, migration and invasion

To investigate the correlation between the expression of Spry1 and other determinants of a malignant phenotype, we next tested both Spry1-silenced and control 1A9 cells for their capability to migrate and invade *in vitro*. Our scratch assay (Figure 3D) indicated that Spry1 knockdown led to a significantly increased percent closure at the 40 h endpoint (p-value: 0.0259). In the migration assay, the Spry1-silenced group showed a significantly higher number of migrated cells 14 (p-value: 0.0125) and 20 hours (p-value: 0.0090) after plating (Figure 3E). The invasion assay (Figure 3F) indicated that 1A9 cell invasion through the matrigel-coated membrane at hours 14 and 20 was significantly higher in the Spry1-silenced group (p-values of 0.0298 and 0.0373, respectively). Taken together, the knocked-down expression of Spry1 resulted in enhanced motility and invasion of 1A9 cells, *in vitro*.

### Induced expression of Spry1 triggers apoptotic events in SKOV-3 cells and inhibits activation of ERK and AKT

**Figure 4:**
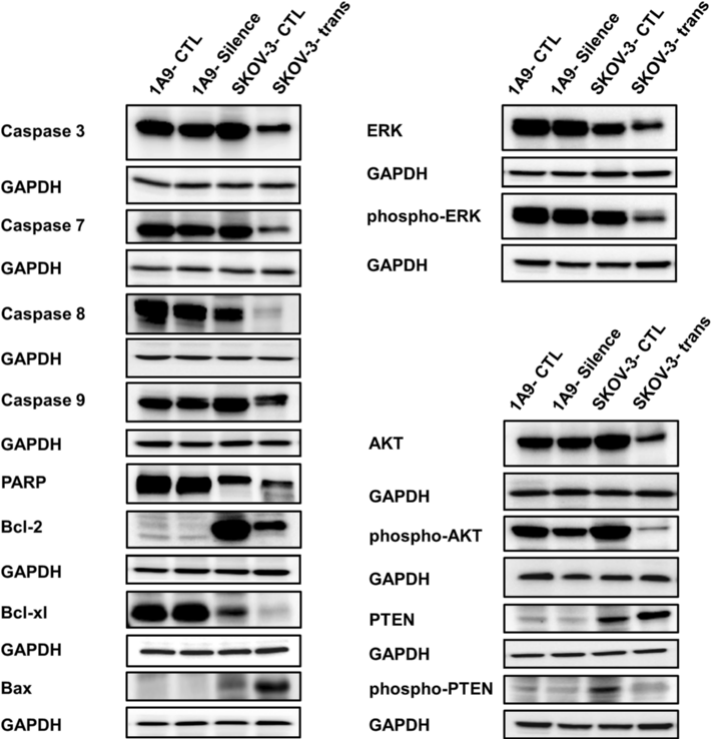
**Western blot analysis of the expression of a number of proteins impacting proliferation and survival of ovarian cancer cells 48 hours after alteration of the Spry1 expression.** Results indicate activation of apoptotic processes detected as overexpression of the pro-apoptotic Bax, decreased expression of the antiapoptotic proteins Bcl-2 and Bcl-xl, attenuation of procaspases 3, 7, 8 and 9 and cleavage of PARP in the Spry1-transfected SKOV-3 cells (left). Moreover, reduced expression of the activated forms of ERK and AKT along with increased expression of PTEN with concomitant decrease of phospho-PTEN (right) implicates repression of ERK as well as of AKT, with involvement of PTEN for the latter, in Spry1-induced inhibition of cell proliferation and survival. No significant change in the expression pattern of these proteins was found in the Spry1-silenced 1A9 cells.

To investigate the implication of apoptotic processes in the Spry1-induced growth inhibition found in this study, the expression of a number of apoptosis-associated proteins in the Spry1-transfected SKOV-3 cells was then examined. As detected by Western blot 48 hours post transfection, overexpression of the pro-apoptotic Bax along with decreased expression of the antiapoptotic proteins Bcl-2 and Bcl-xl were observed. Attenuation of procaspases 3, 7, 8 and 9 as well as cleavage of PARP, an indicator of the caspase 3 activity, was also evident (Figure 4-left). Given the pivotal role of MAPK/ERK and AKT pathways in cell proliferation and survival, we next evaluated the activation status of these pathways after Spry1 transfection. Our results showed that induced expression of Spry1 markedly reduced activated forms of ERK and AKT. Moreover, the expression of phosphatase and tensin homolog (PTEN), a major negative regulator of AKT signaling, was found to be increased. This was accompanied by a concomitant decrease in phospho-PTEN known as the inactive form of cytoplasmic PTEN (Figure 4-right). No significant change in the expression pattern of the above proteins was found in the Spry1-silenced 1A9 cells.

## Discussion

Our previous study revealed that human ovarian cancer cell lines, including SKOV-3 and 1A9, differentially express Spry1. We observed that while the expression of Spry1 was moderately positive in 1A9 cells, it was barely detectable in SKOV-3 cells [8]. Meanwhile, it has been shown that SKOV-3 and 1A9, respectively, exhibit high and low potentials for migration [10] and invasion [11-14]. On this basis, we postulated that the cellular content of the Spry1 protein, a known downstream regulator of RTK, could be a determinant of the ovarian cancer cell behavior. To test our hypothesis, we intended to examine how alteration of the Spry1 expression in SKOV-3 and 1A9 could impact their malignant phenotype assayable by functional tests. To the best of our knowledge, this is the first attempt towards exploring the role of Spry1 in the human epithelial ovarian cancer. Data from the present study demonstrate that while induced expression of Spry1 in the human ovarian cancer cell line with minimal Spry1 content (SKOV-3) attenuates cell proliferation and diminishes survival, knockdown of the protein expression in the Spry1-expressing cell line (1A9) enhances cell viability. Our findings are in line with the results from earlier studies on a number of normal cells. Gross et al. [15] showed that Spry1 inhibits growth and differentiation of NIH3T3 fibroblasts. Spry1 negative regulation of the endothelial cell proliferation has been indicated in HUVEC cells by Impagnatiello et al. [16] and Lee et al. [17]. Using CPAE and ABAE endothelial cells, Huebert et al. [18] and Sabatel et al. [19] have consistently reported Spry1-induced inhibition of the endothelial cell proliferation. Xiang et al. [20] showed that genistein, a phytoestrogen with potential cardioprotective effects, modulates proliferation of quiescent endothelial cells against that of vascular smooth muscle cell (VSMC) through regulating the Spry1 expression. Moreover, Spry1 capability to inhibit growth and proliferation of cancer cells has been explored by some investigators. Kwabi-Adoo et al. [4] reported that overexpression of Spry1 in the prostate cancer cell lines LNCaP and PC3 had an inhibitory effect on colony formation, cell proliferation and viability. In a study by Macia et al. [6], the expression of Spry1 reportedly restrained the proliferation of the human medullary thyroid carcinoma cell line TT *in vitro* and significantly inhibited tumor growth in the murine xenografts. Jin et al. [5] demonstrated that Pokemon- or miR-21-induced suppression of Spry1 stimulated growth and proliferation of the QGY-7703 hepatocellular carcinoma cells while its upregulation inhibited clonogenic growth and proliferation *in vitro*. Sabatel et al. [19] found that the positive Spry1 regulation induced by 16 K prolactin can delay tumor growth in a chimeric mouse model of human colon carcinoma.

Another aspect of the Spry1 function in our study was exhibited when induced expression of Spry1 in SKOV-3 cells attenuated cell motility and, conversely, Spry1 knockdown in 1A9 cells promoted migration and invasion. Our lab has already reported that Spry1 is a partner protein of the urokinase-type plasminogen activator receptor (uPAR) [21] which is able to inhibit uPAR-stimulated migration and invasion in the Saos-2 osteosarcoma, MDA-MB-231 breast cancer and HCT116 colorectal cancer cell lines [22]. Overexpression of Spry1 also inhibited migration of HEK293 human embryonic kidney cells stably transfected with uPAR (HEK293/uPAR). Our results are in agreement with an earlier study on ABAE endothelial cells where partial silencing of Spry1 not only protected ABAE endothelial cells from apoptosis and enhanced cell proliferation, but also promoted cell migration *in vitro*[19].

Exploring mechanisms underlying anti-proliferative and anti-survival effects of the Spry1 transfection in ovarian cancer cells, we found that induced expression of Spry1 activates proapoptotic processes, with implication of Bcl-2 protein family and caspase pathways. Our results also indicate that Spry1 expression inhibits activation of ERK and AKT in SKOV-3 cells. The role of the Sprouty protein family in regulating ERK and AKT stimulation of cell proliferation and survival is well documented [2]. This regulatory function has been studied in a number of cancer cells, including leiomyosarcoma [23], cervical [24], liver [25,26] and breast [27] cancer cells. Moreover, our results implicate PTEN in the Spry1-induced inhibition of AKT where increased amount and activity of PTEN accompanied attenuation of AKT phosphorylation. It has been shown that Sprouty mediates its anti-proliferative effects, at least in part, by increasing the amount and activity of PTEN that in turn attenuates AKT signaling [24]. Polytarchou et al. [28] provided evidence that a hypoxia-activated, Akt-dependent pathway is present in ovarian cancer where the microRNA miR-21 is induced by Akt and subsequently targets and downregulates *Spry1*, *PTEN* and *programmed cell death 4 (PDCD4),* resulting in enhanced cell survival.

Taken together, our results highlight the role of Spry1 in ovarian cancer cell biology. Since such cellular processes as proliferation, migration, invasion, and survival are central to the development, progression, and dissemination of malignant conditions, in-depth understanding of pertinent regulatory mechanisms and their functional significance could lead to the development of novel approaches for enhanced management of cancer. We are currently conducting a retrospective study to investigate clinicopathological relevance of the Sprouty expression profile in patients with ovarian cancer.

## Conclusions

In summary, we report for the first time that the Spry1expression inversely correlates with growth, proliferation, invasion and migration of ovarian cancer cells. Our results suggest that Spry1 may function as a negative regulator of vitality and survival and an inhibitor of motility and invasion in human ovarian cancer cell biology. In other words, the malignant phenotype of the ovarian cancer cell lines might be reflected in part by their ability to differentially express Spry1. Further investigation is underway to elucidate the role of Spry1 and other members of the Sprouty family in ovarian cancer and to evaluate practical value of this protein family in novel diagnostic, prognostic and therapeutic strategies.

## Abbreviations

RTK: Receptor tyrosine kinase; FGF: Fibroblast growth factor1; Spry1: Sprouty protein 1; MTT: Methylthiazol tetrazolium; siRNA: Small interfering RNA; HUVEC: Human umbilical vein endothelial cell; CPAE: Bovine pulmonary artery endothelium; ABAE: Adult Bovine Aortic Endothelial cell; VSMC: Vascular smooth muscle cell; ERMS: Embryonal subtype of rhabdomyosarcoma; uPAR: Urokinase-type plasminogen activator receptor; FBS: Fetal bovine serum; BSA: Bovine serum albumin.

## Competing interests

The authors declare that they have no competing interests.

## Authors’ contributions

SMM designed the study, carried out the experiments, performed data interpretation and statistical analysis and prepared the manuscript. AA contributed to data acquisition and manuscript editing. AQW and AE reviewed manuscript. DLM provided the study concept, contributed to quality control of data and reviewed the manuscript. All authors read and approved the final manuscript.
